# Identification of Multiple Soluble Fe(III) Reductases in Gram-Positive Thermophilic Bacterium *Thermoanaerobacter indiensis* BSB-33

**DOI:** 10.1155/2014/850607

**Published:** 2014-08-07

**Authors:** Subrata Pal

**Affiliations:** Department of Life Science and Biotechnology, Jadavpur University, Kolkata 700 032, India

## Abstract

*Thermoanaerobacter indiensis* BSB-33 has been earlier shown to reduce Fe(III) and Cr(VI) anaerobically at 60°C optimally. Further, the Gram-positive thermophilic bacterium contains Cr(VI) reduction activity in both the membrane and cytoplasm. The soluble fraction prepared from* T. indiensis* cells grown at 60°C was found to contain the majority of Fe(III) reduction activity of the microorganism and produced four distinct bands in nondenaturing Fe(III) reductase activity gel. Proteins from each of these bands were partially purified by chromatography and identified by mass spectrometry (MS) with the help of *T. indiensis* proteome sequences. Two paralogous dihydrolipoamide dehydrogenases (LPDs), thioredoxin reductase (Trx), NADP(H)-nitrite reductase (Ntr), and thioredoxin disulfide reductase (Tdr) were determined to be responsible for Fe(III) reductase activity. Amino acid sequence and three-dimensional (3D) structural similarity analyses of the* T. indiensis* Fe(III) reductases were carried out with Cr(VI) reducing proteins from other bacteria. The two LPDs and Tdr showed very significant sequence and structural identity, respectively, with Cr(VI) reducing dihydrolipoamide dehydrogenase from *Thermus scotoductus* and thioredoxin disulfide reductase from* Desulfovibrio desulfuricans*. It appears that in addition to their iron reducing activity *T. indiensis* LPDs and Tdr are possibly involved in Cr(VI) reduction as well.

## 1. Introduction

The involvement of iron in a number of physiological functions, such as a cofactor for enzymes or an electron acceptor in anaerobic respiration, of most organisms has been recognized [1]. However, most of the metabolic functions of iron require the Fe(II) oxidation state which is more soluble and, therefore, more bioavailable than the Fe(III) form [2].

One of the strategies bacteria have evolved for assimilation and utilization of iron is the reduction of Fe(III) to Fe(II) [3]. Bacterial iron reduction has been studied extensively and various biomolecules responsible for Fe(III) reduction have been discovered [4]. Fe(III) reductases involved in anaerobic respiration are either secreted into the extracellular medium or localized in the cell membrane [5]. Nonetheless, most of the known Fe(III) reductases have been found to be soluble, residing in either the cytoplasm or the periplasmic space [5]. Further, based on the fact that Fe(III) is solubilized by chelators it has been suggested that even soluble Fe(III) reductase could participate in anaerobic respiration [6].

Soluble Fe(III) reductases have been isolated from different bacteria [2–4]. They have been shown to be oxidoreductases transferring electrons from NADH or NADPH to Fe(III) with flavin mononucleotide (FMN), flavin adenine nucleotide (FAD), or riboflavin as the cofactor [3]. They are in fact flavin reductases capable of reducing ferric compounds [7]. In the bacterium* Clostridium kluyveri* an NAD(P)H-dependent flavoenzyme dihydrolipoyl dehydrogenase (synonymously called dihydrolipoamide dehydrogenase) reduces Fe(III) complexes of citrate, ATP, and ADP [2]. In* Thermus scotoductus* SA-01 and* Magnetospirillum gryphiswaldense* MSR-1 thioredoxin reductases, which belong to the family of pyridine nucleotide-disulfide oxidoreductases, reduce soluble Fe(III) [3, 4].

We have earlier reported that a low-GC content Gram-positive* Thermoanaerobacter*-like bacterium (now designated as* Thermoanaerobacter indiensis* BSB-33) reduces Fe(III) and Cr(VI) anaerobically at 60°C optimally [8]. The sequence of* T. indiensis* genome has been determined and annotated (http://www.jgi.doe.gov). Taking advantage of this we have attempted to identify the soluble proteins which are involved in Fe(III) reduction by a combination of chromatography and mass spectrometry (MS). Bioinformatic analysis has also been carried out to find sequence and three-dimensional structural similarity of these proteins with known Cr(VI) reductases.

## 2. Materials and Methods

### 2.1. Strain and Culture

*T. indiensis* BSB-33 (ATCC BAA-2171) cells were grown anaerobically in Luria-Bertani (LB) medium at 60°C in Hungate bottles under 100% N_2_ atmosphere. Transfers and sampling of cultures were carried out anaerobically.

### 2.2. Enzyme Assays

Fe(III) reduction assay was performed by a modification of the procedure of Kaufmann and Lovley [6] under anaerobic conditions at 60°C in a mixture containing 50 mM HEPES (pH 7.0), 1 mM Fe(III)-citrate, 1 mM NADH, 1 mM ferrozine, and 30 to 100 *μ*g of protein. One unit (U) is defined as the amount of enzyme reducing 1 *μ*mol of Fe(III) per min. Cr (VI) reduction assay was carried out anaerobically at 60°C as described earlier [9]. In-gel Fe(III) reduction assay was done by 10% nondenaturing polyacrylamide gel electrophoresis (PAGE) of protein fractions. The gel was stained according to the protocol of Gaspard et al. [10].

### 2.3. Cell Fractionation

Cells were harvested by centrifugation at 400 g for 10 min and washed twice in N_2_-saturated Tris-HCl buffer (pH 7.0) containing 75 mM NaCl, 75 mM KCl, 20 mM MgCl_2_, and 0.1 mM dithiothreitol. 10 g of wet cells was resuspended in 70 mL of the same buffer, 200 *μ*L DNase I (20 mg/mL) and 40 *μ*L RNase I (20 mg/mL) were added, and the suspension was stirred on ice for 1 h. Cells were disrupted by three passages through a French pressure cell at 8,000 psi. The crude lysate was centrifuged at 10,000 ×g for 30 min at 4°C and the supernatant was removed and centrifuged at 100,000 ×g for 1 h at 4°C. The supernatant was used as the soluble fraction whereas the pellet was resuspended in 50 mM Tris-HCl, pH 7.0, to be treated as the membrane fraction. Protein was quantified using Bradford reagent.

### 2.4. Chromatography

The chromatographic procedures followed were modifications of the methods described by Kaufmann and Lovley [6]. The soluble fraction was concentrated by ammonium sulfate precipitation (30 to 80%) and applied to a Superdex 200 prep grade column (1.6 × 60 cm; Amersham Pharmacia Biotech) equilibrated with N_2_-saturated 50 mM Tris-HCl (pH 7.0) containing 75 mM NaCl, 75 mM KCl, 20 mM MgCl_2_, and 2 mM CaCl_2_. Proteins were eluted with the same buffer. The fractions containing Fe(III) reductase activity were pooled, diluted fourfold, and applied to a HiTrap Q HP Sepharose column (1.6 × 2.5 cm; GE Healthcare Life Sciences) equilibrated with 20 mM Tris-HCL (pH 7.5). Bound proteins were eluted with a linear gradient of 0–0.6 M NaCl. The fractions containing maximum Fe(III) reductase activity were pooled, dialyzed in 10 mM sodium phosphate buffer, pH 6.8, and applied to a HT hydroxyapatite column (1 × 15 cm; BioRad) equilibrated with the same buffer. Bound proteins were eluted with a linear gradient of 10–200 mM sodium phosphate buffer, pH 6.8. Active fractions were dialyzed in 20 mM Tris-HCl, pH 7.0, and applied to a mono Q column (0.5 × 5 cm; GE Healthcare Life Sciences) equilibrated with 20 mM Tris-HCl, pH 7.0, and 0.1 M NaCl. Proteins were eluted using a linear gradient of 0.1–0.5 M NaCl in 20 mM Tris-HCl, pH 7.0.

### 2.5. Mass Spectrometry

Protein bands were manually excised from Coomassie-stained nondenaturing gels. MS and protein identification were carried out at Scripps Center for Metabolomics and Mass Spectrometry. Samples were analyzed by ESI-Ion Trap MS. Identification was performed by Mascot software (Matrix Science) searches against a database consisting of* T. indiensis* proteome sequences obtained from http://www.jgi.doe.gov.

### 2.6. Sequence and Structural Similarity

Pairwise sequence similarity was determined using BLASTP (http://blast.ncbi.nlm.nih.gov/Blast.cgi). Amino acid sequences of proteins were aligned using ClustalW2 (http://www.ebi.ac.uk/Tools/msa/clustalw2). Three-dimensional (3D) structures of proteins were built from amino acid sequences using automated homology modeling program ESyPred3D that is based on a very efficient alignment strategy and the MODELLER package to build the final structural model [11, http://www.expasy.org/proteomics/protein_Structure]. 3D structures were compared using DaliLite [12, http://www.ebi.ac.uk/Tools/structure/dalilite] and PDBeFold [13, http://www.ebi.ac.uk/msd-srv/ssm/cgi-bin/ssmserver]. For visualization and superposition of 3D structures UCSF Chimera v.1.8 downloaded from http://www.cgl.ucsf.edu/chimera was used [14]. Functional domains were searched by InterProScan (http://www.ebi.ac.uk/InterproScan).

## 3. Results and Discussion

### 3.1. Localization of Fe(III) Reductase Activity

**Figure 1:**
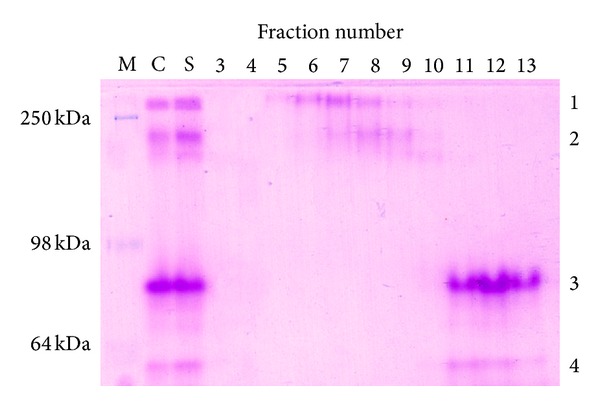
Fe(III) reductase activity gel. 40 *μ*g of proteins each from crude cellular extract (lane 1) and soluble fraction (lane 2) was resolved by 10% nondenaturing PAGE. The soluble fraction concentrated by ammonium sulfate precipitation (30 to 80%) was applied to a Superdex 200 gel filtration column. Three micrograms of proteins each from fractions 3–13 was also electrophoresed in the same gel. Markers (M) are indicated on the left. Fe(III) reductase activity bands are marked on the right.

Crude extracts of the thermophilic bacterium* T. indiensis* reduced Fe(III)-citrate with NADH as the electron donor. The specific activity of reduction measured at 60°C was 0.052 *μ*mol min^−1 ^mg^−1^ protein (Table 1). On fractionation both the soluble and membrane fractions were found to contain Fe(III) reductase activity. However, 76% of the NADH-dependent Fe(III) reductase activity in the crude cell extracts was recovered in the soluble fraction (Table 1). The specific Fe(III) reductase activity in the soluble fraction was also about threefold higher than that in the membrane fraction (Table 1). The soluble extract resolved by nondenaturing PAGE produced four distinct Fe(III) reductase activity bands 1–4 (Figure 1).

In order to identify the protein(s) responsible for each of the Fe(III) reductase activities a four-step chromatographic procedure was followed. The activities were separated from each other by passing sequentially through Superdex 200, Q sepharose, hydroxyapatite, and mono Q columns. The gel filtration step could resolve activities 1 and 2 from activities 3 and 4 (Figure 1). However, in this step activity 1 could not be resolved completely from activity 2 and, similarly, activity 3 could not be separated from activity 4 (Figure 1).

**Figure 2:**
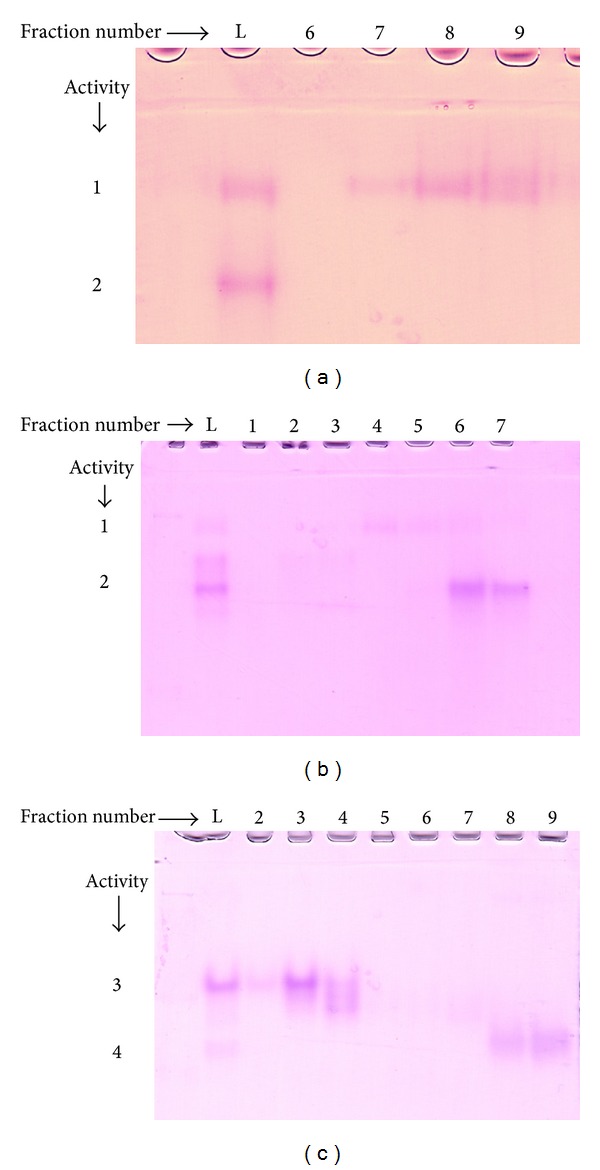
Zymogram analysis of Q Sepharose fractions. Protein samples were resolved by 10% nondenaturing PAGE and visualized by Fe(III) reductase staining. (a) Gel filtration sample 7 was passed through a Q Sepharose column. Four micrograms of proteins from gel filtration fraction 7 (L) and eight micrograms of proteins each from Q Sepharose fractions 6–9 were electrophoresed. (b) Gel filtration fraction 9 was passed through a Q Sepharose column. Eight micrograms of proteins from gel filtration fraction 9 (L) and 15 *μ*g proteins each from Q Sepharose fractions 1–7 were electrophoresed. (c) Gel filtration fraction 12 was passed through a Q Sepharose column. Four micrograms of proteins from gel filtration fraction 12 (L) and 15 *μ*g proteins each from Q Sepharose fractions 2–9 were electrophoresed.

In the second step gel filtration fraction 7 showing a more intense activity 1 band than activity 2 was passed through a Q Sepharose ion-exchange column. Q Sepharose fraction 8 was found to contain the maximum activity 1 free from activity 2 (Figure 2(a)). Similarly, gel filtration fraction 9 which showed greater activity 2 than activity 1 was loaded on a Q Sepharose column. Fraction 6 from Q Sepharose contained activity 2 free from activity 1 as shown in the zymogram (Figure 2(b)). In a similar manner Q Sepharose chromatography could also resolve activity 3 and activity 4 from gel filtration fraction 12 (Figure 2(c)).

In order to purify each of the four activities from nonferric reductase proteins respective Q Sepharose fractions were then passed through hydroxyapatite and mono Q columns. In each step the number of background protein bands was reduced. However, none of the four activities could be completely purified from nonferric reductase protein background (data not shown). Hence, after the mono Q step the protein bands showing Fe(III) reductase activity in preparative nondenaturing gels were excised and analyzed by MS which has the capability to identify multiple proteins from a mixture [15, http://masspec.scripps.edu/services/proteomics/ dataanal.php].

### 3.2. Dihydrolipoamide Dehydrogenase

MS spectra obtained from band 1 matched against two dihydrolipoamide dehydrogenases (LPDs) in* T. indiensis* with high Mascot scores (Table 2). The two proteins contain 469 (LPD1) and 551 (LPD2) amino acid residues, respectively. 3D structures of LPD1 and LPD2 were predicted by EsyPred3D which used* Geobacillus stearothermophilus* dihydrolipoamide dehydrogenase crystal structure (PDB ID 1EBD) as the template (Table 3). High sequence similarity and 3D structural identity were found between the two proteins (Table 4). These are likely to be paralogs having similar functions in the cell.

LPDs belong to pyridine nucleotide-disulfide oxidoreductase family and are found in many species as a part of pyruvate dehydrogenase complex [16]. In the bacterium* Clostridium kluyveri* dihydrolipoyl dehydrogenase (synonymously called dihydrolipoamide dehydrogenase) BfmBC reduces Fe(III) complexes of citrate, ATP, and ADP in an NAD(P)H-dependent manner [2]. It has been proposed that in* C. kluyveri* NADH transfers a hydride ion to enzyme-bound flavin and FADH_2_ thus produced donates an electron to ferric chelates [2].

NCBI BLAST showed 37% and 39% sequence similarity of* C. Kluyveri* BfmBC with* T. indiensis* LPD1 and LPD2, respectively (Table 4). EsyPred3D also predicted the 3D structure of BfmBC using* Geobacillus stearothermophilus* dihydrolipoamide dehydrogenase crystal structure (PDB ID 1EBD) as the template (Table 3). Both LPD1 and LPD2 were found to be identical to BfmBC by 3D structural alignment (Table 4). Further, the two* T. indiensis* proteins as well as* C. kluyveri* BfmBC contain the most conserved FAD-binding sequence motif XhXhGXGXXGXXXhXXh(X)_8_hXhE(D), where X is any residue and h is a hydrophobic residue, as a part of the Rossmann fold [17]. Hence, it was expected that like the* C. kluyveri* enzyme either of the* T. indiensis* LPDs would be able to reduce Fe(III) complexes which is consistent with the zymogram result (Figure 1).

### 3.3. Thioredoxin Reductase

Among the high-scoring matches obtained by MS of the protein mixture from band 2 the only* T. indiensis* protein likely to possess Fe(III) reductase activity is thioredoxin reductase (Trx). Thioredoxin reductase-like ferric reductase (FeS) as well as typical thioredoxin reductase (Trx) is also present in* T. scotoductus* SA-01 [4]. Both* T. scotoductus* enzymes can reduce Fe(III)-NTA complex although reduction by Trx is less efficient [4].* Magnetospirillum gryphiswaldense* MSR-1 thioredoxin reductase FeR5 has also been shown to possess ferric reductase activity [3].* T. indiensis* Trx showed 29% sequence similarity (92% query coverage) with* T. scotoductus* Trx and 24% sequence similarity (95% query coverage) with* M. gryphiswaldense* FeR5 by BLAST analysis (Table 4). ESyPred3D program predicted the 3D structures of* T. indiensis* Trx and* M. gryphiswaldense* FeR5 by using* Escherichia coli* thioredoxin reductase crystal structure (PDB ID 1CL0) as the template and the 3D structure of* T. scotoductus* Trx by using* Deinococcus radiodurans* thioredoxin reductase crystal structure (PDB ID 2Q7V) as the template (Table 3). PDBeFold and DaliLite analyses also found significant structural identity of* T. indiensis* Trx with* T. scotoductus* Trx as well as* M. gryphiswaldense* FeR5 (Table 4). In spite of such structural similarity with other thioredoxin reductases,* T. indiensis* Trx lacks the disulfide redox centre CXXC. It may be questioned if this enzyme could at all reduce thioredoxin and be called a thioredoxin reductase. However, InterProScan showed that it contains a FAD/NAD(P)-binding domain. It could possibly catalyze electron transfer from NAD(P)H to FAD and the reduced flavin could, in turn, reduce Fe(III) substrate [4]. In fact, in case of* T. scotoductus* FeS absence of CXXC has been found to be advantageous with respect to Fe(III) reduction [4].* T. indiensis* Trx also appears to be a thioredoxin reductase-like ferric reductase.

### 3.4. NAD(P)H-Nitrite Reductase

MS with samples extracted from band 3 showed the presence of* T. indiensis* NAD(P)H-nitrite reductase which was the only protein in the mixture likely to be an Fe(III) reductase. InterProScan identified a FAD/NAD(P)-binding domain in the protein and suggested that it belonged to pyridine nucleotide-disulfide oxidoreductase family of proteins. BLAST analysis found significant sequence similarity of* T. indiensis* nitrite reductase to NAD(P)H-nitrite reductases from* Clostridium tetani*,* Bacillus cereus*, and* Heliobacterium modesticaldum* (data not shown). Domain analysis showed that all these nitrite reductases belonged to the pyridine nucleotide-disulfide oxidoreductase family. A search in the whole PDB archive found* Pyrococcus furiosus* nitrite reductase to be structurally similar to* T. indiensis* nitrite reductase (PDBeFold *Z* = 11.64, rmsd = 2.16). It is not known if any of these pyridine nucleotide-disulfide oxidoreductases could reduce iron. However, as described earlier, one pyridine nucleotide-disulfide oxidoreductase,* C. kluyveri* dihydrolipoamide dehydrogenase BfmBC, has been shown to reduce Fe(III) complexes of citrate, ATP, and ADP in an NAD(P)H-dependent manner [2]. The 3D structure of* T. indiensis* nitrite reductase was predicted by ESyPred#D using* Novosphingobium aromaticivorans* ferredoxin reductase ArR crystal structure (PDB ID 3LXD) as the template (Table 3). Sequence and structural similarity of* T. indiensis* nitrite reductase with* C. kluyveri* BfmBC were found to be moderate (Table 4). Hence, it is likely that* T. indiensis* NAD(P)H-nitrite reductase is responsible for the reduction of Fe(III)-nitrilotriacetic acid in the native gel as shown in Figure 1.

### 3.5. Thioredoxin Disulfide Reductase

Finally, as identified with high Mascot score, the only* T. indiensis* protein in band 4 most likely to be a Fe(III) reductase is thioredoxin disulfide reductase (Tdr) (Table 2). BLAST analysis found high sequence similarity of* T. indiensis* Tdr with* T. scotoductus* Trx and* M. gryphiswaldense* FeR5 (Table 4). The 3D structure of* T. indiensis* Tdr was predicted by ESyPred3D using* Mycobacterium tuberculosis* thioredoxin reductase crystal structure (PDB ID 2A87) as the template (Table 3). 3D structural alignment of* T. indiensis* Tdr against* T. scotoductus* Trx and* M. gryphiswaldense* FeR5 carried out with both PDBeFold as well as DaliLite produced significant values (Table 4). Besides, like the other thioredoxin reductases, FAD and NAD(P)H binding domain of* T. indiensis* Tdr also contained redox-active site CATCD. Hence, the enzyme possesses the true characteristics of a thioredoxin reductase. In fact,* T. indiensis* Tdr has a greater sequence and structural similarity to* T. scotoductus* Trx and* M. gryphiswaldense* FeR5 than* T. indiensis* Trx. Therefore, it can be surmised that like the* T. scotoductus* and* M. gryphiswaldense* enzymes* T. indiensis* Tdr also reduced Fe(III) as evident from the zymogram (Figure 1).

*T. indiensis* Tdr also showed 43% similarity (97% query coverage) with a thioredoxin reductase (TR) from the hyperthermophilic bacterium* Thermotoga maritima* [18]. Three-dimensional structure of* T. maritima* was predicted using* Salmonella enteric* AhpF crystal structure (PDB ID 1HYU) as the template (Table 3). PDBeFold analysis also showed significant structural alignment between the two enzymes (*Z* = 15.95 and rmsd = 1.90). Both enzymes have a typical FAD-binding motif GXGXXG near the N-terminus, a nicotinamide nucleotide-binding motif GGGXXA near the middle, and an active redox center CXXC [18]. Like thermophilic* T. indiensis* BSB-33,* T. maritima* is capable of growing as a respiratory organism when Fe(III) is provided as an electron acceptor and* T. maritima* cell suspension reduces Fe(III) [19]. However, whether* T. maritima* TR is involved in Fe(III) reduction has not yet been shown.

### 3.6. Similarity with Cr(VI) Reductases

It has been reported earlier that like several other mesophilic and thermophilic microorganisms* T. indiensis* BSB-33 is capable of reducing Cr(VI) besides Fe(III) [8]. In fact, some bacterial proteins have demonstrated their capability to reduce both Fe(III) and Cr(VI) [20, 21]. In* Shewanella oneidensis* outer membrane proteins MtrC and OmcA are involved in both Fe(III) and Cr(VI) reduction [20]. However, to the best of our knowledge, the only cytoplasmic enzyme that has been shown to reduce both Fe(III) and Cr(VI) is FerB from* Paracoccus denitrificans* [21]. Initially FerB was characterized as a ferric reductase [21]. Subsequently, it was found to be capable of reducing chromate as well and showed significant sequence similarity with known chromate reducers ChrR from* Pseudomonas putida* and YieF from* Escherichia coli* [21].

**Figure 3:**
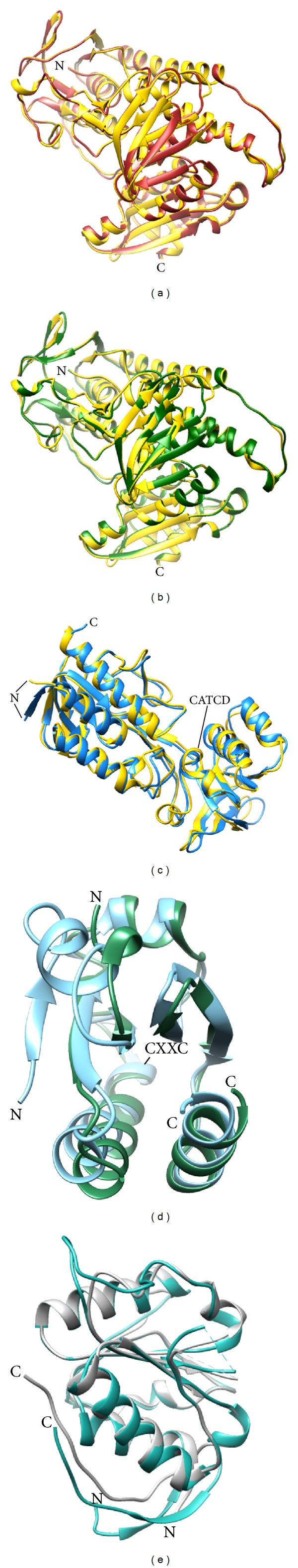
Pairwise structural superposition of* T. indiensis* proteins with Cr(VI) reduction proteins of different bacteria. (a)* T. indiensis* LPD1 (brown) with* T. scotoductus* LPD (gold); (b)* T. indiensis* LPD2 (green) with* T. scotoductus* LPD (gold); (c)* T. indiensis Tdr* (gold) with* D. desulfuricans* MreE (blue); (d)* T. indiensis* thioredoxin (blue) with* D. desulfuricans* thioredoxin (green); (e)* T. indiensis* indolepyruvate ferredoxin oxidoreductase (blue) with* D. desulfuricans* MreG (grey). N-termini, C-termini, and redox-active CATCD and CXXC have been indicated.

In* Thermus scotoductus* SA-01 a peripheral membrane-associated protein, identified as a dihydrolipoamide dehydrogenase (LPD) by sequence homology, has been shown to couple NAD(P)H oxidation to Cr(VI) reduction [16]. The enzyme contains a redox-active disulfide which is not present in ChrR [16]. Both* T. indiensis* LPDs were found to possess significant sequence similarity with the* T. scotoductus* protein (Table 5).* T. scotoductus* LPD 3D structure was also predicted using* Geobacillus stearothermophilus* dihydrolipoamide dehydrogenase crystal structure (PDB ID 1EBD) as the template (Table 3). Structural alignments of* T. indiensis* LPDs with* T. scotoductus* LPD gave high *Z*-score and rmsd (Table 5) and there was also considerable overlap between their 3D structures (Figures 3(a) and 3(b)). Multiple sequence alignment by ClustalW2 also identified in* T. scotoductus* LPD as well as* T. indiensis* LPDs FAD- and NAD(P)-binding Rossmann folds amino acid sequence motif GX_1-2_GXXG together with either GXXXG or GXXXA motifs where the first glycyl residue of these motifs and the third glycyl residue of the GX_1-2_GXXG motif are the same [22]. It is, therefore, quite likely that both* T. indiensis* LPDs would also reduce Cr(VI) in an NAD(P)H-dependent manner.

Further, in* D. desulfuricans* a thioredoxin reductase MreD has been shown to be involved in NAD(P)H-dependent reduction of U(VI) and Cr(VI) in association with thioredoxin and possibly an oxidoreductase protein MreG [23]. The reduction mechanism as indicated consists of transfer of electrons from NAD(P)H to thioredoxin catalyzed by thioredoxin reductase [23]. Reduced thioredoxin could then transfer the electrons directly to oxidized metals or indirectly through MreG [23]. Three-dimensional structures of* D. desulfuricans* MreD, thioredoxin, and MreG and corresponding proteins of* T. indiensis* were predicted by ESyPred3D using PDB templates as enlisted in Table 3.

*T. indiensis* thioredoxin was found to contain the typical thioredoxin fold which is a distinct structural motif consisting of a four-stranded *β*-sheet and three flanking *α*-helices [24]. ClustalW2 alignment has shown that it also shared a CXXC active site motif. Structural alignment of* T. indiensis* and* D. desulfuricans* thioredoxins gave *Z* = 9.10, rmsd = 1.597 (PDBeFold) and *Z* = 13.5, rmsd = 1.7 (DaliLite).* T. indiensis* also contained a protein indolepyruvate ferredoxin oxidoreductase which was structurally similar to* Desulfovibrio* pyruvate ferredoxin/flavodoxin oxidoreductase MreG (PDBeFold: *Z* = 10.17, rmsd = 1.19; DaliLite: *Z* = 25.1, rmsd = 1.6).

Sequence similarity between* T. indiensis* Tdr and* D. desulfuricans* MreE was found to be 37% with 97% query coverage. Structural identity between the two enzymes as shown by *Z*-score, rmsd, and superposition was very high (Table 5; Figure 3(c)). Besides, like the other thioredoxin reductases,* T. indiensis* Tdr also contained a redox-active site CATCD. Hence, based on the overall sequence and structural similarity, it could be inferred that like the thioredoxin-thioredoxin reductase system of* D. desulfuricans*, the thioredoxin-thioredoxin system of* T. indiensis* could also reduce Cr(VI). It is also to be noted that neither* T. indiensis* Tdr nor LPDs showed any significant sequence or structural match with ChrR, YieF, or FerB indicating the possibility of different chromate reduction mechanisms in this bacterium.

## 4. Conclusion

In conclusion, the soluble fraction of* T. indiensis* contains four distinct Fe(III) reductase activities. The annotated genome sequence of the thermophilic bacterium has enabled identification of the proteins responsible for Fe(III) reductase activity by chromatography and mass spectrometry. Two of these activities are due to pyridine nucleotide-disulfide family of enzymes, namely, two paralogous dihydrolipoamide dehydrogenases and NAD(P)H-nitrite reductase. A thioredoxin reductase-like protein is responsible for the third activity whereas the fourth activity is caused by a thioredoxin disulfide reductase having all the characteristics of a true thioredoxin reductase. Besides, dihydrolipoamide dehydrogenases and thioredoxin-thioredoxin reductase system of* T. indiensis* are also possibly responsible for chromate reductase activity of the bacterium. The genome sequence of* T. indiensis* will enable cloning and overproduction of the reductases identified in this work and further analysis to elucidate the similarities and differences of iron and chromium reduction pathways.

## Figures and Tables

**Table 1:** Localization of Fe(III) reductase activity.

Fraction	Total protein recovered (mg)	Specific activity (U/mg)	Total activity (U)	% of activity in crude extract recovered
Crude extracts	880.5	0.052	45.8	100
Soluble fraction	609.8	0.057	34.8	76
Membrane fraction	95.9	0.02	1.9	4

One U (unit) is defined as the amount of enzyme reducing 1 *µ*mol of Fe(III) per min.

**Table 2:** Fe(III) reductases identified by MS analysis of active bands in zymogram gels.

Gel band	Reductase	Jgi id	Sequence coverage (%)	Peptides match	Score
1	Dihydrolipoamide dehydrogenase	2517398608	43	19	1135
	Dihydrolipoamide dehydrogenase	2517398836	32	17	994
2	Thioredoxin reductase	2517400540	38	15	1034
3	NAD(P)H-nitrite reductase	2517399369	46	16	895
4	Thioredoxin disulfide reductase	2517400648	43	10	784

**Table 3:** PDB templates used by ESyPred3D program for three-dimensional structure prediction of proteins.

Bacteria/target proteins	Templates (PDB ID)
*T. indiensis *	
LPD1	*Geobacillus stearothermophilus* dihydrolipoamide dehydrogenase (1EBD)
LPD2	*Geobacillus stearothermophilus* dihydrolipoamide dehydrogenase (1EBD)
Trx	*Escherichia coli* thioredoxin reductase (1CL0)
NAD(P)H-nitrite reductase	*Novosphingobium aromaticivorans* ferredoxin reductase ArR (3LXD)
Tdr	*Mycobacterium tuberculosis* thioredoxin reductase (2A87)
Thioredoxin	Hyperstable thioredoxin from Precambrian period (2YJ7)
Indolepyruvate ferredoxin oxidoreductase	*Pyrococcus furiosus* 2-keto acid:ferredoxin oxidoreductase subunit alpha (1YD7)
*C. kluyveri *	
BfmBC	*Geobacillus stearothermophilus* dihydrolipoamide dehydrogenase (1EBD)
*T. scotoductus *	
Trx	*Deinococcus radiodurans* thioredoxin reductase (2Q7V)
LPD	*Geobacillus stearothermophilus* dihydrolipoamide dehydrogenase (1EBD)
*M. gryphiswaldense *	
FeR5	*Escherichia coli* thioredoxin reductase (1CL0)
*D. desulfuricans *	
MreE	*Escherichia coli* thioredoxin reductase (1CL0)
Thioredoxin	*Desulfovibrio vulgaris* Hildenborough thioredoxin (2L6C)
MreG	*Pyrococcus furiosus* 2-keto acid:ferredoxin oxidoreductase subunit alpha (1YD7)
*T. maritima *	
TR	*Salmonella enterica* alkylhydroperoxide reductase subunit F AhpF (1HYU)

**Table 4:** Pairwise sequence and structural similarity of *T. indiensis* proteins with Fe(III) reductases.

Protein pair	NCBI BLAST		PDBeFold		DaliLite	
	Query coverage (%)	Identity (%)	*Z*	RMSD∗ (Å)	*Z*	RMSD∗ (Å)
Band 1						
*T. indiensis* LPD1 versus LPD2	97	93	26.07	0.589	60.5	0.6
*T. indiensis* LPD1 versus *C. kluyveri* BfmBC	99	37	25.89	0.459	59.8	0.5
*T. indiensis* LPD2 versus *C. Kluyveri* BfmBC	99	39	25.06	0.582	59.6	0.6
Band 2						
*T. indiensis* Trx versus *T. scotoductus* Trx	92	29	13.96	1.96	36.8	2.3
*T. indiensis* Trx versus *M. gryphiswaldense *FeR5	95	24	18.12	0.949	42.8	1.3
Band 3						
*T. indiensis* NAD(P)H-nitrite reductase versus *C. kluyveri* BfmBC	68	28	10.58	2.46	27.7	3.3
Band 4						
*T. indiensis* Tdr versus *T. scotoductus *Trx	98	51	18.02	1.118	41.7	1.2
*T. indiensis* Tdr versus *M. gryphiswaldense *FeR5	98	46	15.65	1.46	41.3	1.6

∗RMSD: root mean square distance.

**Table 5:** Pairwise sequence and structural similarity of *T. indiensis* proteins with Cr(VI) reductases.

Protein pair	NCBI BLAST		PDBeFold		DaliLite	
	Query coverage (%)	Identity (%)	*Z*	RMSD∗ (Å)	*Z*	RMSD∗ (Å)
Dihydrolipoamide dehydrogenase						
*T. indiensis* LPD1 versus *T. scotoductus* LPD	99	36	26.26	0.54	59.4	0.6
*T. indiensis* LPD2 versus *T. scotoductus* LPD	98	36	25.90	0.78	59.4	0.5
Thioredoxin-thioredoxin disulfide reductase system						
*T. indiensis* Tdr versus *D. desulfuricans *MreE	97	37	16.10	1.33	40.6	1.4
*T. indiensis* thioredoxin versus *D. desulfuricans* thioredoxin	69	27	9.10	1.60	13.5	1.7
*T. indiensis* indolepyruvate ferredoxin oxidoreductase versus *D. desulfuricans* MreG	75	32	10.17	1.19	25.1	1.6

∗RMSD: root mean square distance.
